# Supplementary material for: Parity-independent Kondo effect of correlated electrons in electrostatically defined ZnO quantum dots
Source: Nat Commun. 2024 Nov 7;15:9556. doi: 10.1038/s41467-024-53890-2 (PMC11543668; doi:10.1038/s41467-024-53890-2)
Supplement: Supplementary file 1 — Supplementary Information [file 41467_2024_53890_MOESM1_ESM.pdf]

Supplementary Information: Parity-independent Kondo effect of correlated electrons in electrostatically defined ZnO quantum dots

Kosuke Noro,[1,2] Yusuke Kozuka,[3] Kazuma Matsumura,[1,2] Takeshi Kumasaka,[1] Yoshihiro Fujiwara,[1,2] Atsushi Tsukazaki,[4,5] Masashi Kawasaki,[6,7] and Tomohiro Otsuka[1,2,5,7,8,*]

[1]*Research Institute of Electrical Communication, Tohoku University, 2-1-1 Katahira, Aoba-ku, Sendai 980-8577, Japan*

[2]*Department of Electronic Engineering, Graduate School of Engineering, Tohoku University, 6-6 Aramaki Aza Aoba, Aoba-ku, Sendai 980-0845, Japan*

[3]*Research Center for Materials Nanoarchitechtonics (MANA), National Institute for Material Science (NIMS), 1-1 Namiki, Tsukuba 305-0044, Japan*

[4]*Institute for Materials Research, Tohoku University, 2-1-1 Katahira, Aoba-ku, Sendai 980–8577, Japan*

[5]*Center for Science and Innovation in Spintronics, Tohoku University, 2-1-1 Katahira, Aoba-ku, Sendai 980-8577, Japan*

[6]*Department of Applied Physics and Quantum-Phase Electronics Center (QPEC), University of Tokyo, 7-3-1 Hongo, Bunkyo-ku, Tokyo 113-8656, Japan*

[7]*Center for Emergent Matter Science, RIKEN, 2-1 Hirosawa, Wako, Saitama 351-0198, Japan*

[8]*WPI Advanced Institute for Materials Research, Tohoku University, 2-1-1 Katahira, Aoba-ku, Sendai 980-8577, Japan*

(Dated: October 1, 2024)

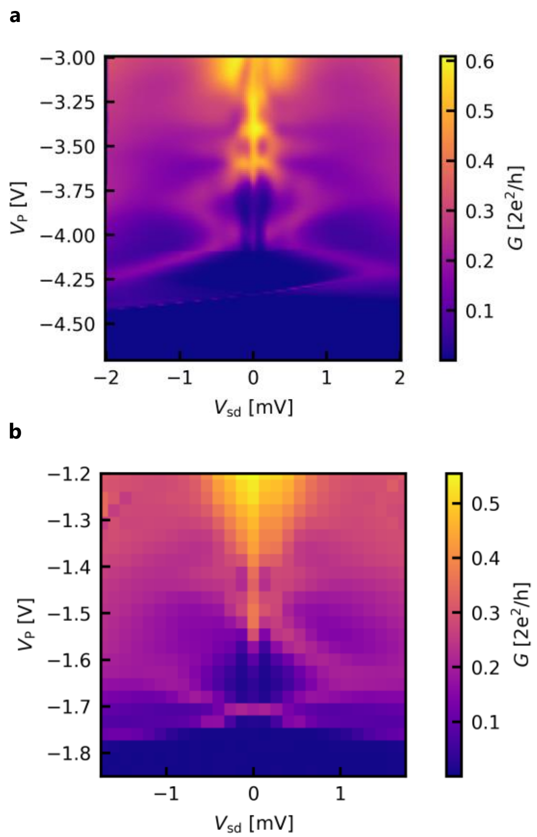

Supplementary Figure 1. **a**, **b** Conductance map as functions of V_{sd} and V_P in different devices.

KONDO EFFECTS OBSERVED IN DIFFERENT DEVICES

Supplementary Figure 1a and b show the conductance (G) maps as functions of the source-drain bias V_{sd} and the gate voltage V_P in quantum dot devices different from that used in the main text. We can see the zero-bias Kondo peaks in these devices, which illustrate the robustness of the Kondo effects in ZnO quantum dots. The breakdown of the even-odd electron parity effects is also observed in different devices.

* tomohiro.otsuka@tohoku.ac.jp
